# Impact of early parenteral nutrition completing enteral nutrition in adult critically ill patients (EPaNIC trial): a study protocol and statistical analysis plan for a randomized controlled trial

**DOI:** 10.1186/1745-6215-12-21

**Published:** 2011-01-24

**Authors:** Michaël P Casaer, Greet Hermans, Alexander Wilmer, Greet Van den Berghe

**Affiliations:** 1 Department of Intensive Care Medicine, University Hospitals Leuven, Katholieke Universiteit Leuven, Belgium; 2 Medical Intensive Care Unit, Department of General Internal Medicine, University Hospitals Leuven, Katholieke Universiteit Leuven, Belgium

## Abstract

**Background:**

For critically ill patients treated in intensive care units (ICU), two feeding strategies are currently being advocated, one by American/Canadian and the other by European expert guidelines. These guidelines differ particularly in the timing of initiating parenteral nutrition (PN) in patients for whom enteral nutrition (EN) does not reach caloric targets.

**Methods/Design:**

The EPaNIC trial is an investigator-initiated, non-commercial, multi-center, randomized, controlled, clinical trial with a parallel group design. This study compares early (European guideline) versus late (American/Canadian guideline) initiation of PN when EN fails to reach a caloric target. In the early PN group, PN is initiated within 24-48 hours after ICU admission to complete early enteral nutrition (EN) up to a calculated nutritional target. In the late PN group, PN completing EN is initiated when the target is not reached on day 8. In both groups, the same early EN protocol is applied. The study is designed to compare clinical outcome (morbidity and mortality) in the 2 study arms as well as to address several mechanistical questions. We here describe the EPaNIC study protocol and the statistical analysis plan for the primary report of the clinical results.

**Discussion:**

The study has been initiated as planned on august 01 2007. One interim analysis advised continuation of the trial. The study will be completed in February 2011.

**Trial Registration:**

ClinicalTrials (NCT): NCT00512122

## Background

### Nutritional support in ICU patients

It remains unclear whether artificial nutritional support beneficially affects outcome of critically ill patients. The route of administration, the delay before initiating such artificial nutrition, the amount of calories and possibly also the type of nutrient formula may be of importance [1-3].

As compared to parenteral nutrition (PN), enteral nutrition (EN), and early EN in particular, has been reported to be associated with less (infectious) complications [4-6], lower risk for pronounced hyperglycemia [5] and thus EN could be safer as well as cheaper. However, relying solely on EN often results in not achieving the caloric targets [7-9]. Indeed, even in stable intensive care unit (ICU) patients, early initiation of EN was associated with a high incidence of gastrointestinal intolerance and serious adverse events, such as regurgitation, suspected aspiration or colectasia, which necessitated stopping of EN [10]. Even after formal implementation of an evidence-based nutrition protocol in the ICU, the mean time to administering enteral feeding appeared to be about 3 days [1]. Furthermore, the mean percentage of caloric target achieved by day 4 was below 70% [1]. In addition, as physicians are unable to accurately predict which patient in the ICU will be resuming normal oral or enteral nutrition within one week after admission, the risk of underfeeding critically ill patients during the first week in ICU is substantial [11]. Indeed in many patients, solely relying upon EN has shown to result in underfeeding [7,8]. Underfeeding has been associated with an increased incidence of infection [12], and with other complications such as prolonged ventilation, prolonged length of stay and pressure ulcers [13,14]. In spite of such association between malnutrition and adverse outcomes, it has not been investigated in adequately powered randomized clinical trials (RCT) whether parenterally completing failing EN early in the course of critical illness provides an outcome benefit.

Combining PN with EN indeed represents a strategy to prevent such malnutrition, but has frequently led to overfeeding [7,15]. Overfeeding has been associated with increased risk of infection and metabolic disturbances, such as hyperglycemia, dyslipidaemia and liver dysfunction, as well as with prolonged mechanical ventilation [16,17]. Such a risk of overfeeding, however, could be prevented by using a computer-assisted feeding protocol via the patient data management systems (PDMS) currently in use in ICUs, as such system could remind physicians to reduce the PN supplement proportionally to the increase in enteral intake. Furthermore, a meta-analysis demonstrated that elevated blood glucose levels with early PN as compared with early EN may at least partially explain more infectious and non-infectious complications [5,18,19].

Skeletal muscle weakness in ICU patients is a serious threat for failed weaning from mechanical ventilation and for hampered rehabilitation. Typically, about one third of prolonged critically ill patients develop critical illness polyneuropathy and/or myopathy resulting in important neuromuscular weakness [20]. Theoretically, preventing starvation early during critical illness may partially prevent skeletal muscle catabolism and hereby prevent substantial muscle wasting. However, aggressive nutrition did not effectively reduce muscle breakdown and instead resulted in fat accumulation [21]. Part of the failure to reduce muscle catabolism with PN could be related to the concomitant hyperglycemia as recently shown in an animal model [22,23]. It thus remains unclear whether prevention of hyperglycemia with insulin during early provision of optimal amounts of calories with a combination of EN plus PN, could prevent such complications [15].

### Conflicting guidelines and practices

Current American/Canadian as well as European clinical practice guidelines for nutritional support in the critically ill strongly recommend that EN be used in preference to PN whenever the gastrointestinal tract is intact and functional.

The American/Canadian guidelines in addition recommend that PN should *not *be started at the same time as EN. Also, it is advised that hypocaloric nutrition should be tolerated during the first week in ICU for patients who are not malnourished prior to ICU admission [24,25].

The recent European Society for Parenteral and Enteral Nutrition guidelines for parenteral nutrition in intensive care recommend the administration of supplemental parenteral nutrition within 2 days after ICU admission to patients who cannot be fed sufficiently via the enteral route [26]. Such expert opinion is in part explained by recent data from meta-analyses that revealed a lower mortality with PN in critically ill patients [6].

Current guidelines for nutritional support of patients in the ICU therefore differ substantially among continents and countries. Such differences are explained by absence of high level evidence for either of the strategies applied worldwide and therefore the guidelines are all largely based upon expert opinion. These differences in nutritional practices not only indicate the lack of evidence for impact on patient outcome but also represent important differences in costs for critical care [9,27,28].

### Rationale of the study

In view of the 2 different practice guidelines widely adopted based upon strong expert opinion but in the absence of high level evidence, there is need for an adequately powered randomized controlled trial comparing the two nutritional practices.

**Figure 1:**
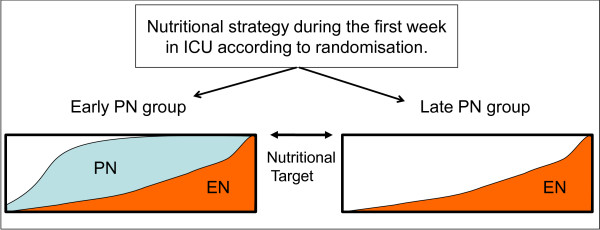
**Conceptual cartoon of study design**.

In a context of prevention of hyperglycemia and while avoiding overfeeding, a strategy based on early PN completing EN up to a calculated caloric and protein target (further referred to as "early PN") could be superior to withholding PN during the first week of critical illness (further referred to as "late PN") by preventing weakness and enhancing recovery. Alternatively, withholding PN during the first week of critical illness ("late PN") while advocating early EN may shorten ICU and hospital stay by avoiding complications of early PN. Finally, clinical equivalence of the 2 feeding strategies would generate evidence in favor of withholding PN for one week in ICU in view of the cost of this intervention. (Figure 1)

In this randomized controlled study which has the acronym "EPaNIC", we compare "early PN" with "late PN" in ICU patients at risk of developing malnutrition in the ICU. The primary focus of the EPaNIC study is clinical outcome, more specifically the dependency on intensive medical care and need for hospitalization as well as vital status. In addition, a series of mechanistic studies as well as a long-term follow-up is planned. The latter however do not form the basis of this manuscript.

## Methods/Design

### Patient eligibility for inclusion and recruitment

Upon admission to ICU, all adult patients undergo nutritional screening with the Nutritional Risk Screening (NRS 2002) score. This is a scoring system developed to detect the presence of malnutrition and the risk of developing malnutrition in the hospital [29].

All adult patients admitted to any of the 7 participating intensive care units, who present with an NRS score higher or equal than 3, are eligible for inclusion in the EPaNIC study. The following patients are not considered eligible for inclusion: patients with a "do not resuscitate" code at the time of ICU admission, patients expected to die within 12 hours, patients readmitted to ICU after randomization to the EPaNIC trial, patients enrolled in another trial, patients transferred from another ICU after a stay of more than seven days, and patients suffering from ketoacidotic or hyperosmolar coma on admission. Moreover, patients with a body mass index (BMI) <17 (kg/m2), patients with short bowel syndrome treated with home-PN, patients on home mechanical ventilation, pregnant or lactating women are not included. Finally, patients without a clinical indication or with a contra-indication for a central venous catheter and patients who are still able to take oral nutrition on ICU admission or with an NRS score lower than 3 are excluded from this trial.

### Ethical aspects and informed consent

Written informed consent is obtained from the patient or the closest family member or legal guardian. The patient or the next of kin can withdraw from the study at any time, without penalty or impact on treatment.

A register is kept of all patients evaluated for inclusion and of patients who withdraw from the study. The latter are clinically followed up without their data being analyzed in the study.

The study protocol and consent forms were approved by the Institutional Review Board (IRB) of the Katholieke Universiteit Leuven, University Hospitals (approval number ML4190) and by the competent Belgian authorities (EudraCT 2007-000169-40). The Jessa Hospitals' Institutional Review Board IRB (Hasselt) gave positive advice for the addition of 2 of their ICUs (Unit A and C) at the study site Jessa Hospitals.

### Data collected at study entry

At baseline, data on demographic and clinical characteristics of the patients are obtained. Disease specific risk scores are calculated and co-morbidities and known use of important medications prior to admission are noted: these comprise, among others, Acute Physiology and Chronic Health Evaluation II score (APACHE II), presence of cancer, diabetes mellitus, kidney failure (Chronic Kidney Disease score), liver failure (Child classification), COPD (Gold classification), and heart failure (New York Heart Association Functional Classification ≥ 2). Sepsis upon admission is labeled as such using modified Bone criteria [30,31].

### Randomization

The study has a prospective, randomized, controlled, parallel-group design. Consecutive patients are randomly assigned to one of the two treatment study groups, labeled "Early PN" or "Late PN", using sequentially numbered, sealed and opaque envelopes. Upon addition of the study site Jessa Hospitals, these envelopes were replaced by an identical digital system allowing central computerized randomization. Patients are stratified per study site according to 17 primary diagnostic categories on admission. The stratified groups were labeled as follows:

I Medical-ICU admissions: (a) respiratory; (b) cardiovascular; (c) renal; (d) hematological/oncological; (e) GI/hepatic; (f) metabolic; (g) neurological (h) other;

II Surgical-ICU admissions according to referral discipline (a) cardiac surgery (elective or urgent surgery) (b) complicated thoracic surgery; (c) complicated vascular surgery (d) complicated abdominal and pelvic surgery (e) complicated neurosurgery (f) trauma and burns (g) transplantation (h) neurological disease (i) other.

Randomization - in a one to one allocation ratio - was performed using permuted blocks of 10, information that remained unknown to bed-side physicians and nurses, responsible for patient recruitment and therapy assignment.

#### a. Randomized interventions

##### "Early PN"

Patients randomized to the "early PN" group receive Glucose 20% at 40 ml/hr on the admission day. PN [OliClinomel^® ^or - when fat free PN is indicated - Clinimix^® ^(Baxter, Brussels Belgium)] is initiated the second morning in ICU. The amount of PN to be given on any particular day is the difference between calculated caloric needs and the calories delivered by EN the previous 24 hours. Caloric needs calculations are based on corrected ideal body weight, age and gender [32] moreover, we defined an absolute maximal target of 2880 kcal/day. When EN covers 80% of calculated caloric needs, PN is stopped. When the patient is able to eat, the PN is reduced and eventually stopped. Whenever enteral or oral intake falls below 50% of calculated caloric needs, the PN is restarted.

##### "Late PN"

Patients randomized to the "late PN" group receive a volume of Glucose 5% that is required to obtain adequate hydration taking into account the volume of EN that is being delivered. If enteral feeding of at least 80% of the calculated calories is not possible after 7 days in ICU, PN is initiated on day 8.

#### b. Common strategy for early EN in both study arms

In all patients unable to eat on the second evening of ICU stay and without formal contraindication, EN is initiated with the patients in semi-recumbent position. The increase of EN volume, the use of gastroprokinetics and duodenal feeding tubes are described in the standing-orders for EN. Parenteral trace elements, minerals and vitamins are administered in all patients of both groups as clinically indicated.

In both study groups, blood glucose levels are targeted to 80-110 mg/dl with continuous insulin infusion [18]. Blood glucose and potassium are monitored every 1 - 4 hours on the bloodgas-analyzer (ABL-Radiometer^®^) using arterial blood samples.

The volumes of PN and EN to be given according to the treatment group are calculated by the PDMS (Metavision^®^, iMDsoft, Boston, USA). These calculations are based on the nutritional intake during the previous day and the clinical evolution of the patient. Patients are weaned from the ventilator according to a standardized guideline used in all participating ICUs. End-of-care decisions in patients for whom further intensive care is considered to be futile are taken in consensus by a group of two senior ICU physicians and the referring specialist.

#### Handling of re-admissions to ICU

Patients who are re-admitted to ICU after a participation in EPaNIC are not eligible for re-inclusion. Patients who are readmitted to the ICU within 48 hours of discharge and who are still within the 7 days time window of the initial randomization receive the nutrition-schedule they were assigned to during the initial ICU admission. Patients readmitted later will be fed at the discretion of the attending physician.

#### Blinding of treatment allocation

Treating physicians and patients could obviously not be blinded. However, all outcome assessors, which are investigators not directly involved in the patients care (such as statisticians, laboratory personnel, infectious disease specialists, pathologists, physiotherapists involved in the strength measurement, electrophysiologists) as well as physicians and nurses in the conventional wards, are blinded to treatment allocation.

#### Data collection following recruitment

All medications received by the patients during ICU stay are registered. Every day the amount of kilocalories, lipids, proteins, carbohydrates delivered by either PN or EN are calculated from the PDMS in an automated manner and entered into the case report form (CRF). For calculation of the energy requirements, 50% of the gastric residual volume, which is being discarded by the bedside nurses, is considered to be EN calories not absorbed by the patient. Interruptions of EN delivery and predefined digestive intolerance are registered daily. Mechanical complications such as displacement or obstruction of the enteral feeding tube or the central venous catheters; and clinical complications such as pneumothorax, hemothorax and subclavian or carotid artery puncture are recorded daily. Number of ICU days with a central line in situ is also noted. In addition we record the need for and the number of days of mechanical ventilatory support, of mechanical and pharmacological hemodynamic support, of renal replacement therapies and placement of tracheostomy.

Blood samples (a subset of which are immediately stored on ice for future endocrinological measurements) are taken upon ICU admission and daily at 06:00 h until discharge from ICU or death. All whole blood glucose levels are measured on arterial blood using a blood gas analyzer on each ICU and are registered for later calculation of glucose metrics.

Analyses on blood and urine for the primary clinical analyses include routine chemistry, hematology, and markers of inflammation. Further metabolic, endocrine and inflammatory measurements planned on stored samples in the context of mechanistic analyses fall beyond the scope of this paper. In addition, for later mechanistic studies, a random selection of patients are prospectively and repeatedly screened for the presence of sludge or cholecystitis on ultrasonography.

All new infections of the lungs, the blood stream, the urinary tract and wounds are recorded by an infectious disease specialist. Bacteraemia is further classified by responsible pathogen and as catheter related blood stream infection versus other bacteraemia [33,34].

For further mechanistical and exploratory studies, elaborated muscle strength testing, electrophysiological examination, skeletal muscle and adipose tissue biopsies and radiological evaluation of skeletal muscle and adipose tissue compartments are performed after specific or additional informed consent from the patient or the legal guardian. These analyses will be repeated at different time intervals, also after hospital discharge, on condition of obtaining adequate additional funding. The statistical plan for all these analyses fall beyond the scope of this manuscript. Furthermore, as far as practically feasible, every patient is approached just prior to hospital discharge, for a 6 minute walking distance test (6 MWD) [35] by a trained physiotherapist and for scoring the activity in daily life (ADL). The long-term follow-up will include sending out a Medical Outcomes Study 36 items short form questionnaire to all patients.

#### Data conservation and processing

Data are collected electronically in an anonymized CRF, unambiguously linked to the source file. Data is manually transferred (and checked for accuracy) into the CRF by the clinical research assistance team on a daily basis from the ICU PDMS and the Leuven University Hospitals Clinical Working Station (KWS). Extensive range and consistency checks are performed by the study monitor. Vital status at 90 days will be recorded for all patients, eventually via the Belgian National Registry when this information is not available in the hospital information system.

## Trial Organisation

### Administrative and legal aspects

The sponsor (K.U.Leuven) provides direct access to the CRF, the source data and the study master file for monitoring, Independent Ethics committee review and regulatory inspection. The sponsor established an independent data safety monitoring board (DSMB) (RB, PL and JV). The sponsor appointed one monitor (PW). The monitor verifies that the trial is performed in accordance to the protocol as described in the European Medicine Agency's "Note for guidance on good clinical practice CPMP/ICH/135/95." as well as the Declaration of Helsinki. Monitoring is performed and reported following the sponsor's standing operating procedures. No fault insurance is covered by Fortis Corporate Insurance NV.

### Trial Coordination

The clinical research team guarantees a daily follow up of patient screening and inclusion, availability of requested clinical data in the clinical patient files and protocol compliance. Every non-compliance to the protocol and other questions or problems are reported to the study monitor (PW) and discussed with the principal investigators. Serious Adverse Events (SAE) are reported to the study sponsor (K.U.Leuven). The study monitor (PW) regularly provides the sponsor with reports on inclusions and SAE. Regular meetings are organized with principal investigators and clinical research team to discuss the daily progression of the research project.

### Protocol implementation

**Figure 2:**
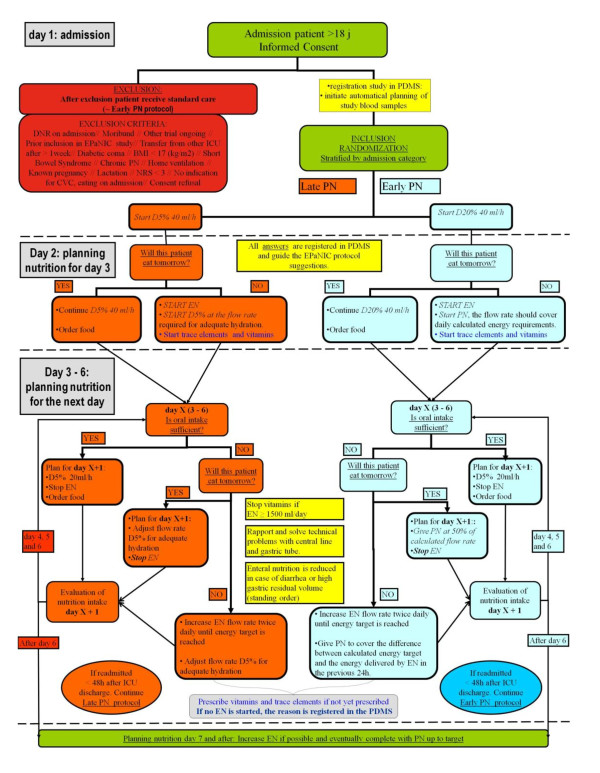
**Trial procedures flow sheet**.

The protocol was and is being instructed (by MC) to all clinical medical and paramedical staff trough frequent teaching sessions, clinical feedback rounds and posters representing the study flow. (Figure 2) The protocol decision support is integrated into the ICU patient data management system, facilitating the prescription of the exact amounts of PN and EN according to protocol and clinical evolution. (WDB, DC, GM and MC)

### Interim analysis

As both the nutritional strategies investigated in this trial are current ICU practices, the DSMB judged that repeated interim analyses for efficacy were not required. One formal "safety" interim analysis was planned after patient number 1500 left the ICU, during which the independent DSMB had access to un-blinded results on ICU mortality, hospital mortality and serious adverse events from 1495 patients. The DSMB judged that there was no reason to prematurely stop the clinical trial for safety reasons, or to perform more safety interim analyses and decided that the randomization and nutritional therapy study had been executed as planned in the study protocol. The DSMB advised to continue the study to completion, under monitoring of the serious adverse events. Because the primary efficacy endpoint was not analyzed, no correction of the significance level at the final analysis is necessary.

## Statistical analysis plan

We here report the statistical analysis plan for the primary clinical report of the primary and secondary clinical endpoints of this RCT. These include the acute clinical effects of the intervention during ICU stay and hospitalization, including survival up to 90 days after randomization. Additional long-term outcomes, further mechanistic and exploratory analyses of the effect of early PN versus late PN will be reported separately and statistical details for these additional studies fall beyond the scope of this manuscript. The additional outcomes comprise, among others, results of elaborated peripheral and respiratory muscle testing and electrophysiological signs of myopathy-neuropathy, radiographic and microscopic evaluation of muscle and adipose tissue volumes and composition, biochemical and molecular analyses of skeletal muscle and adipose tissue biopsies, endocrine function, liver and bile function. Furthermore, a health economy analysis is planned.

### General rules of the statistical analyses

**Figure 3:**
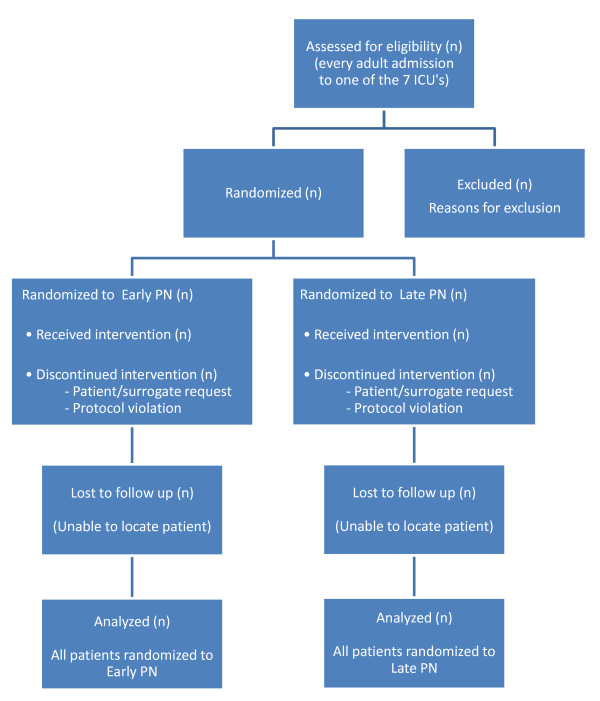
**CONSORT flow diagram**.

A consort diagram will be reported. (Figure 3) All analyses will be performed on a full intention-to-treat basis. The data file will be finalized 90 days after inclusion of the last patient.

To assess compliance with the study protocol, the amounts of PN and EN actually given in the two study groups during the intervention window of 8 days will be reported as absolute numbers and percentages of target calories.

Discrete variables will be summarized by frequencies and percentages. Continuous variables will be summarized by use of either mean or standard deviations (SD) or median and interquartile range as appropriate.

Baseline and outcome variables will be compared using Student's t-test, (exact) Chi-square test and Mann-Whitney-U test, as appropriate.

All outcomes will be analyzed in an uncorrected manner as well as jointly corrected for risk factors (type and severity of illness, age, BMI and NRS categories). Type of illness will comprise the diagnostic categories, as stratified and grouped per organ system, as well as the presence or absence of a history of cancer. As severity of illness score for this correction, APACHE II will be used. BMI will be categorized as <20, 20-<25, 25-<30, 30-<40 and ≥40 kg/m2. Applicable NRS categories for this study are 3, 4, 5, 6 and 7. In case certain categories would appear underrepresented, such patients will be assigned to the next category in rank or previous category if it is the last category that is underrepresented.

A priori defined subgroup analyses will be performed for BMI and NRS subcategories. These subgroup analyses are based on the rationale that BMI has been reported to be associated with different risk of outcome in different types of ICU patients which may be due to different handling of nutritional substrates [36-38]. This risk appeared different for patients with overweight or obesity (BMI 25 to <40) versus all other BMI categories (undernourished and normal BMI [BMI <20 and BMI 20-<25] or morbid obesity [BMI ≥ 40]). Therefore BMI will be dichotomized as such to optimize the power of this subgroup analysis. Also patients at higher risk of malnutrition may respond differently to the feeding strategies. Therefore, in this subgroup analysis, NRS will be dichotomized separating the highest risk patients [NRS ≥ 5] from the moderate risk patients [NRS 3 and 4]. Another a priori defined subgroup analysis will be performed for patients admitted to ICU after emergency or elective cardiac surgery as compared with all other patients. The shorter time in ICU of this subgroup may result in the early PN group receiving predominantly extra glucose 20% infusion, without lipids and amino-acids, as compared with the glucose 5% in the late PN group, which may evoke a different response than the full week combined parenteral nutrition. As sepsis is known to aggravate the metabolic disturbances evoked by early PN, a priori subgroup analysis is planned for patients with and without sepsis upon admission. These a priori defined subgroup analyses will be performed for the primary and safety endpoints without correction for other variables. To test for interaction between the identified subgroups and the studied intervention, a multivariable analysis (logistic regression or proportional hazard analysis as appropriate) will be performed for each subgroup variable separately and with the subgroup variable, the intervention and their interaction in the model. The interaction will be tested at a significance level of 0.1.

The first clinical report will analyze the impact of the nutritional strategies on safety (among which mortality), primary and secondary efficacy endpoints.

For all endpoints, differences will be considered statistically significant whenever the p-value reaches 0.05 or lower without correction for multiple testing.

### Safety endpoints

Safety endpoints comprise vital status (mortality 90 days after randomization independent of ICU and hospital discharge status, hospital mortality, ICU mortality and proportion of patients discharged alive from ICU within 8 days), hypoglycemia, serious adverse events and complications related to the mode of nutrition.

Survival up to 90 days after randomization in both treatment groups will be compared by Kaplan Meier survival plots. The impact of "late PN" versus "early PN" will be analyzed, with and without correction for age, BMI & NRS categories and type and severity of illness, by Cox proportional hazard analysis. Vital status up to 90 days predictably will be traceable for virtually all patients via the National Registry, loss to follow-up will likely not be present except for some patients who will have a residence outside the Belgian territory. In addition, we will record vital status at ICU and hospital discharge and 90 days after randomization, and will analyze differences with Chi-square testing. All analyses will be performed in the intention to treat population. Correction of such differences in ICU and hospital mortality for age, BMI & NRS categories and type and severity of illness, will be performed using a multivariable logistic regression analysis, on condition of the absence of co-linearity between risk factors. Frequencies and percentages for both study arms will be presented together with odds ratios and 95% confidence intervals. As the randomized study intervention only takes place during a time window up to the 8^th ^day in ICU, we also plan to analyze early lethality within this time window in the intention to treat population as part of the safety analysis.

As patients not receiving early PN may be considered at increased risk for hypoglycemia, we will report for both groups the number of patients experiencing hypoglycemia <40 mg/dl during the time window of the randomized intervention. Hypoglycemia resistant to parenteral glucose administration is considered as a SAE and the incidence during the time window of the randomized intervention will be reported for both groups. In addition, overall blood glucose control during the time window of the intervention will be compared using daily morning blood glucose as well as daily maximal and minimal blood glucose. More sophisticated glucose metrics will be analyzed (among others hyperglycemic index, hypoglycemic index en glycemic penalty index) but such metrics fall beyond the scope of this initial report. Also, requirement of insulin will be compared.

Safety issues also comprise the occurrence of feeding-mode related complications during the time window of the intervention. Therefore, occurrence of these complications (digestive intolerance, complicated insertion of feeding tubes, pneumothorax, hemothorax and subclavian or carotid artery puncture, occlusion or displacement of central venous catheters or gastric feeding tubes) will be reported for both treatment groups.

### Primary efficacy endpoint

The primary efficacy endpoint for this RCT is the time to discharge alive from ICU. As the time of ICU discharge to the regular ward may be affected by the availability of beds on the regular wards, which could induce bias, we a priori decided to analyze "time to discharge from ICU" as "time to ready for discharge from ICU". A patient is considered "ready for discharge" as soon as all clinical conditions for ICU discharge have been fulfilled (no longer in need for vital organ support and receiving at least 2/3 of the caloric requirements as oral feeds) or earlier when the patient is actually sent to a regular ward.

Time to discharge alive from ICU will be reported by Kaplan Meier plots, with ICU non-survivors censored beyond the longest ICU stay of survivors and censoring time of patients still in the ICU at closing of the datafile (90 days after last patient inclusion) over both treatment groups. The impact of "late PN" versus "early PN" will be analyzed, with and without correction for age, BMI & NRS categories and type and severity of illness, by Cox proportional hazard analysis. The distribution of the actual time to discharge from ICU will be reported for ICU-survivors and ICU-non-survivors separately. In view of the time window of the randomized intervention in ICU, also the proportion of patients staying beyond 8 days in ICU will be reported.

### Secondary efficacy endpoints

All analyses will be performed uncorrected as well as corrected for age, BMI & NRS categories type and severity of illness. Time to event analysis will be analyzed similarly as the primary endpoint. Proportion of patients requiring support of vital organ functions and distribution of duration of support will be analyzed by non-parametric or parametric testing depending on the normality of the distribution in the subgroup of patients for which support was needed. Proportions will be compared using chi-square testing. Results of repeated measurements will be analyzed using an appropriate model for longitudinal data.

a. Time to discharge alive from the hospital, with patients still in the hospital at closing of the data file censored at that time point; patients transferred to another hospital censored on the day of transfer; and non-survivors censored beyond the longest hospital stay of survivors and censoring time of patients still in the hospital at closing of the data file (90 days after last patient inclusion) over both treatment groups.

b. Time to final (alive) weaning from mechanical respiratory support with patients still on mechanical respiratory support at closing of the data file being censored at that time point and ICU non-survivors censored beyond the longest duration of mechanical respiratory support of the survivors and censoring time of patients still on mechanical respiratory support at closing of the data file (90 days after last patient inclusion) over both treatment groups.

c. Kidney failure: Proportion of patients in need for renal replacement therapy (RRT) during ICU stay; distribution of duration of RRT (for those patients requiring RRT); proportion of patients with a post-randomization diagnosis of new kidney injury/failure (defined by modified Risk, Injury, Failure, Loss, and End-stage Kidney (RIFLE) classification criteria as a plasma creatinine doubling or more during ICU stay) in both treatment groups.

d. Need for pharmacological or mechanical hemodynamic support during ICU stay, and duration of such need. In addition, time to final (alive) weaning from all pharmacological or mechanical hemodynamic support in ICU will be analyzed, with ICU non-survivors censored beyond the longest duration of pharmacological or mechanical hemodynamic support of the survivors and censoring time of patients still on such support at closing of the data file (90 days after last patient inclusion) over both treatment groups.

e. Need for a tracheostomy during ICU stay.

f. Occurrence of infections during ICU stay: Number of patients with new infections and types of infection as specified in the study protocol and the duration of any antibiotics therapy initiated after randomization for those patients requiring antibiotics.

g. Cholestasis and liver dysfunction: Proportion of patients during the time window of the intervention and during the whole ICU stay presenting with cholestasis, defined as total bilirubinemia above 3 mg/dl or an increase to 150% of baseline value of gamma-glutamyltransferase or alkaline phosphatase. An increase of alanine or aspartate transaminases higher than 3 times the upper limit of normal will be considered as a marker of liver cytolytic damage. Proportion of patients presenting with such liver cytolytic damage during the time window of the intervention and during the whole stay in ICU will be compared. More subtle changes in liver enzymes will be analyzed by comparing the distribution of the highest values and the time course of the daily measurements.

h. Inflammation: Effect of the intervention on inflammation will be analyzed by comparing the distribution of the highest value reached during ICU stay and changes from baseline to the highest value and by comparing time profiles of daily C-Reactive Protein values.

i. Distribution of 6 MWD at hospital discharge in both treatment groups will be compared, as well as the proportion of patients unable to perform the test due to clinical reasons.

j. Proportion of patients independent for all ADL functions in both groups will be compared at hospital discharge. Hospital non-survivors will be analyzed as fully dependent for all functions.

### Sample size calculation

The sample size was calculated in order to detect, with at least 80% power and 95% certainty, an increase or decrease in average duration of ICU stay with one day [from a baseline assumed ICU stay of 8 ± 13 (mean ± SD) days and a median of 4 days assuming log-normal distribution; power calculated using one-tailed Student's t-test using the mean and SD for an increase and a decrease with 1 day and confirmed with two-tailed Student's t-test after log transformation]. To concomitantly detect with at least 70% power and 95% certainty an ICU mortality increase or decrease of 3% (assuming a baseline mortality of 20% anticipating a Medical-ICU/Surgical-ICU patients fraction of 25/75%; power calculated with two-tailed chi-square testing), a sample of 4640 (patients 2320 per arm) was considered necessary. We plan to calculate the true power of the study for detection of any eventual smaller difference in these outcomes.

## Discussion

The study has been initiated as planned on august 01 2007. One interim analysis advised continuation of the trial. The study will be completed in February 2011. A significant difference in the safety and/or efficacy endpoints will provide important evidence for optimizing clinical patient care. Also a neutral result will provide important insight, as this would mean that clinicians can safely withhold PN in all comparable patients during the first week of ICU stay, which has an impact on expenses for critical care.

## Abbreviations

6MWD: 6MWD; ADL: Activities of Daily Life; APACHE: Acute Physiology And Chronic Health Evaluation; BMI: Body Mass Index; CRF: Case Report Form; DSMB: Data Safety Monitoring Board; EN: Enteral Nutrition; ICU: Intensive Care Unit; IRB: Institutional Review Board; KWS: Clinical Working Station; NRS: Nutritional Risk Screening; PDMS: Patient Data Management System; PN: Parenteral Nutrition; RCT: Randomized Clinical Trial; RRT: Renal Replacement Therapy; SD: Standard Deviation.

## Competing interests

The majority of the funding for this trial was provided by the Methusalem program of the Flemish government (via K.U.Leuven to GVdB), the Research Fund of the K.U.Leuven (GOA-2007/14 to GVdB), and the Fund for Scientific Research Flanders, Belgium (FWO doctoral fellowship to MC and FWO post-doctoral fellowship to GH). In addition, K.U.Leuven received an institutional partial (< 30%), unconditional and non-restrictive research grant for this trial from Baxter Healthcare USA. The sponsors had no influence on the design of protocol, patient recruitment, data generation and will not have any impact on the analysis of the results or writing of the manuscript. IP eventually generated from this RCT will be property of the University (K.U.Leuven). The authors have no financial or non-financial competing interests to declare.

The authors received the international "Stoutenbeek Award" 2009 for the quality of the EPaNIC study protocol.

## Authors' contributions

MPC, GH, AW and GVdB all contributed to the design of the EPaNIC study protocol. MPC and GVdB developed the statistical plan, which was approved by all the authors, and drafted the manuscript. All authors read and approved the final manuscript. For the primary report of the clinical results, the authorship will be determined by intellectual and operational contribution (including the number of patients enrolled in each subunit) concordant the Vancouver declaration.
